# Development of a single-dose Q fever vaccine with an injectable nanoparticle-loaded hydrogel: effect of sustained co-delivery of antigen and adjuvant

**DOI:** 10.1080/10717544.2025.2476144

**Published:** 2025-05-02

**Authors:** Lu Wang, Aaron Ramirez, Jiin Felgner, Enya Li, Jenny E. Hernandez-Davies, Anthony E. Gregory, Philip L. Felgner, Ali Mohraz, D. Huw Davies, Szu-Wen Wang

**Affiliations:** a Department of Chemical and Biomolecular Engineering, University of California, Irvine, CA, USA; b Vaccine Research and Development Center, Department of Physiology and Biophysics, University of California, Irvine, CA, USA; c Institute for Immunology, University of California, Irvine, CA, USA; d Department of Biomedical Engineering, University of California, Irvine, CA, USA; e Chao Family Comprehensive Cancer Center, University of California, Irvine, CA, USA

**Keywords:** Nanoparticle vaccine, hydrogel, co-delivery, sustained delivery, Q fever

## Abstract

Q fever is a zoonotic infectious disease caused by *Coxiella burnetii,* and there is currently no FDA-approved vaccine for human use. The whole-cell inactivated vaccine Q-VAX, which is only licensed in Australia, has a risk of causing severe adverse reactions, making subunit vaccines a good alternative. However, most subunit antigens are weak immunogens and require two or more immunizations to elicit an adequate level of immunity. We hypothesized that by combining a nanoparticle to co-deliver both a protein antigen and an adjuvant, together with a hydrogel depot for sustained-release kinetics, a single-administration of a nanoparticle-loaded hydrogel vaccine could elicit a strong and durable immune response. We synthesized and characterized a protein nanoparticle (CBU-CpG-E2) that co-delivered the immunodominant protein antigen CBU1910 (CBU) from *C. burnetii* and the adjuvant CpG1826 (CpG). For sustained release, we examined different mixtures of PLGA-PEG-PLGA (PPP) polymers and identified a PPP solution that was injectable at room temperature, formed a hydrogel at physiological temperature, and continuously released protein for 8 weeks *in vivo*. Single-dose vaccine formulations were administered to mice, and IgG, IgG1, and IgG2c levels were determined over time. The vaccine combining both the CBU-CpG-E2 nanoparticles and the PPP hydrogel elicited a stronger and more durable humoral immune response than the soluble bolus nanoparticle vaccines (without hydrogel) and the free antigen and free adjuvant-loaded hydrogel vaccines (without nanoparticles), and it yielded a balanced IgG2c/IgG1 response. This study demonstrates the potential advantages of using this modular PPP hydrogel/nanoparticle system to elicit improved immune responses against infectious pathogens.

## Graphical abstract

**Figure:**
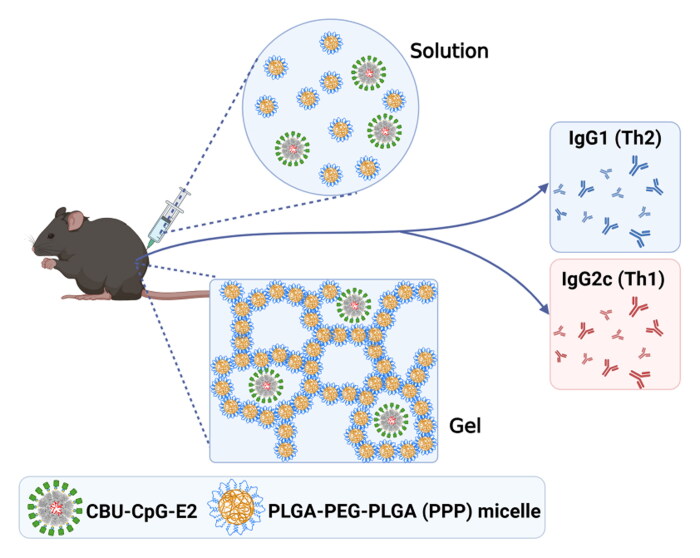

Schematic representation of *in vivo* hydrogel depot vaccine formulation and resulting humoral immune responses

## Introduction

Q fever is a zoonotic infectious disease caused by the Gram-negative bacterium *Coxiella burnetii* (Angelakis and Raoult 2010; van Schaik et al. 2013; Eldin et al. 2017). It has been classified as a biosafety level 3 (BSL-3) pathogen and a Tier 2 Select Agent by the CDC due to its high infectivity, aerosol transmissibility, and resistance to many common disinfection methods (Anderson et al. 2013; Centers for Disease Control and Prevention and HHS 2017; Cherry et al. 2022). In the most notable Q fever epidemic which happened in the Netherlands, 4026 human cases were reported between 2007 and 2010 (Schneeberger et al. 2014), and approximately 1 to 5% of the infected individuals were affected by chronic Q fever, a more severe form of the disease that can be lethal (Kampschreur et al. 2014; Schneeberger et al. 2014; van Roeden et al. 2019). There are two phase variations of *C. burnetii*, virulent phase I and avirulent phase II cells (Sam et al. 2023). Phase I variants express full-length lipopolysaccharide (LPS), whereas phase II variants contains truncated LPS without the terminal O-antigen sugars (Hackstadt et al. 1985). Whole cell vaccines that have been successful at reducing the incidence of disease are derived from *C. burnetii* in phase I, but culturing large volumes of a BSL3 Select Agent presents a significant biosafety and biosecurity challenge to vaccine manufacturers. Livestock vaccination has been implemented as a preventative and control method to avoid spread and economic losses. For example, Coxevac^®^ (CEVA Santé Animale, Libourne, France) is licensed for goats, cattle, and sheep. A recent study evaluated the humoral response of Coxevac^®^ in buffalo under field conditions (Ferrara et al. 2024). A sustained Th1-CD8^+^-type immunity and an increased protection in *C. burnetii*-challenged goats was obtained when adjuvating Coxevac^®^ with QuilA^®^ (Tomaiuolo et al. 2023). Despite Q fever being a highly infectious disease and threat to public health, the only commercial vaccine for human use, Q-VAX, is licensed only in Australia but not available worldwide because of its reactogenic nature (Sam et al. 2023). Furthermore, as a whole-cell inactivated vaccine, prescreening is necessary for Q-VAX due to risk of serious adverse reaction for previously sensitized individuals (Ruiz and Wolfe 2014; Redden et al. 2023). Thus, new vaccines without the highly reactogenic components need to be developed. Subunit vaccines are a safer alternative to whole-cell vaccines since they contain purified antigenic components without other reactogenic components.

Challenges in developing successful protein subunit vaccines, however, are reduced immunogenicity and relatively short half-lives *in vivo*, compared with whole cell vaccines (Hansson et al. 2000; Moyle and Toth 2013; Tsoras and Champion 2019; Butkovich et al. 2021; Nguyen and Tolia 2021). The usage of a nanoparticle (NP) platform to deliver constituents of subunit vaccines helps to improve the immune response to the antigen; the NP scaffold increases both the amount of antigen uptake by antigen presenting cells (APCs) and its drainage to the lymph nodes (Manolova et al. 2008; Irvine and Read 2020; Butkovich et al. 2021). Furthermore, the activated APCs can elicit cytotoxic CD8^+^ T lymphocyte responses that are critical to eradicate intracellular infections (Fan and Moon 2017), such as *C. burnetii* (Sam et al. 2023). In our previous studies, we demonstrated that a protein NP platform, which is derived from the E2 subunit of the pyruvate dehydrogenase complex from *Geobacillus stearothermophilus* (E2), is a thermostable protein nanocage that can be recombinantly engineered for conjugation with immune-activating adjuvants (with introduced cysteines in the internal cavity) and to peptide or protein antigens (on the exterior surface) (Dalmau et al. 2008; Molino et al. 2013, 2016; Neek et al. 2020, 2018; Li et al. 2022; Butkovich et al. 2023; Ramirez et al. 2023). This co-delivery of antigens and adjuvants using the E2 scaffold elicits higher immune responses and has been utilized for the development of vaccines for cancer and infectious diseases. Recently, we synthesized a Q fever vaccine using this NP strategy, which incorporated an immunodominant protein antigen of *C. burnetii* (CBU) (Ramirez et al. 2023) and an adjuvant (CpG), and this elicited a significantly higher humoral immune response than the soluble antigen alone (with no NP) using a two-immunization (prime and booster) schedule.

Although most subunit vaccines require a prime injection and one or more booster administrations to elicit adequate levels of immune response and protection, single-dose vaccines have captured great interest due to advantages such as decreased costs associated with multi-dose immunization, increased global accessibility, and higher levels of patient compliance (Schiller and Lowy 2015; Lofano et al. 2020). However, typical single-dose vaccines are made with a live attenuated form of the target pathogen, which can bring risk to immunocompromised individuals (Lofano et al. 2020). Another route for single-dose vaccines is to encapsulate antigens in polymer-based particles (Preis and Langer 1979; Walters et al. 2015); however, the structural integrity of the antigen can be damaged by the organic solvents used and the manufacturing processes (Walters et al. 2015; Lofano et al. 2020).

Multiple studies have shown that modulating the antigen release kinetics, and more specifically, prolonging antigen exposure, leads to a stronger antigen-specific immune response (Johansen et al. 2008; Tam et al. 2016; Pauthner et al. 2017; Cirelli et al. 2019); this is likely due to sustained antigen exposure which enhances the follicular helper T cell proliferation and maintenance (Baumjohann et al. 2013; Tam et al. 2016; Pauthner et al. 2017; Cirelli et al. 2019), as well as the affinity maturation of B cells (Pauthner et al. 2017; Cirelli et al. 2019). Many sustained delivery systems, including osmotic pumps (Pauthner et al. 2017; Cirelli et al. 2019), PLGA microspheres (Guarecuco et al. 2018; Sarmadi et al. 2022), microneedles (DeMuth et al. 2014; Bian et al. 2022), and hydrogels (Gale et al. 2021; Chen et al. 2022; Nkanga et al. 2022), have been examined to obtain extended antigen exposure times.

In this work, we investigated the sustained release of vaccines by using a thermoresponsive hydrogel. Injectable hydrogels have been studied as drug and vaccine delivery systems to encapsulate a variety of peptides, proteins, nucleic acid, and cells (Lei et al. 2022) with high efficiency. Hydrogels can also be designed to recruit immune cells, such as APCs (Lei et al. 2022), by forming a local inflammatory site (Korupalli et al. 2019; Wang et al. 2019; Roth et al. 2020), providing a niche to host immune cells (Phan et al. 2019, 2022), or releasing cytokine payloads (Kim et al. 2015; Fenton et al. 2019) to increase the vaccine efficacy. Shear-thinning hydrogels have been shown to naturally recover their viscoelasticity after injection (Gale et al. 2021; Fan et al. 2023). Other strategies to form a gel network after *in vivo* administration include *in situ* chemical (Bos et al. 2004; He et al. 2021; Kim et al. 2021) or physical crosslinking (Korupalli et al. 2019; Le et al. 2019), by means of external triggers (Le et al. 2019; Lee et al. 2019; Phan et al. 2019; Nkanga et al. 2022).

The class of polymers we used is poly(lactic-*co*-glycolic acid)–poly(ethylene glycol)–poly(lactic-*co*-glycolic acid) (PLGA-PEG-PLGA). PLGA-PEG-PLGA (PPP) is a triblock copolymer which can form micelles in aqueous solutions at low temperatures and self-assembles to form a hydrogel above its transition temperature (Khorshid et al. 2016; Cui et al. 2019). Both PLGA and PEG are FDA-approved materials for clinical use, and the PPP copolymers are also biocompatible and biodegradable (Yu et al. 2010; Su et al. 2021). PPP hydrogels have been applied to the controlled release of biotherapeutics, including small molecule drugs (Chen et al. 2020, 2021; Sharma et al. 2021), peptides (Li et al. 2013; Liu et al. 2017; Dutta et al. 2020), proteins (Gong et al. 2020; Wei et al. 2021; Yi et al. 2023), and oligonucleotides (Gong et al. 2015; Gao et al. 2020). In previous studies, PPP has also been used as a depot material for the model antigen ovalbumin (OVA) (Wang et al. 2017) and a DNA vaccine against Newcastle disease (Gao et al. 2020). However, to our best knowledge, it has not been used in NP-based subunit vaccines or protein assemblies to improve vaccine immunogenicity.

In this study, we explored the feasibility and effectiveness of using PPP thermosensitive hydrogels to slowly release a NP-derived vaccine which can co-deliver subunit antigens and activating adjuvants. Although other hydrogel vaccine depots have been used to deliver free antigen and adjuvant simultaneously (Gale et al. 2021) or NP-conjugated antigen with (Ou et al. 2023) and without free adjuvants (Nkanga et al. 2022), the efficacy of formulating a hydrogel depot with a dual antigen- and adjuvant-conjugated NP vaccine has not been evaluated. Co-delivering antigen and adjuvant to the same dendritic cell (DC) can help with the activation of DCs (Molino et al. 2013) and avoid immunologic tolerance induced by delivering antigen in the absence of adjuvant (Goldberg 2015). Furthermore, the precise orientation and repetitive array of the antigen protein on the surface of the NP is advantageous for B cell activation and antibody development (Nguyen and Tolia 2021; Ramirez et al. 2023).

Our study therefore investigated the combined immune effects of a NP delivery system together with a PPP hydrogel depot for sustained delivery and serves as a proof-of-principle study of the nanoparticle/hydrogel vaccine delivery platform for its feasibility and function. We examined the model protein antigen CBU1910 (CBU), which is an immunodominant antigen for *C. burnetii* and relevant for a Q fever vaccine. The combined NP + PPP delivery system is modular and allows for vaccine applications toward other infectious agents. The results obtained here could inform the development of other single-dose vaccines.

## Materials and methods

### Materials

Poly(lactic-*co*-glycolic acid)–*b*-poly(ethylene glycol)–*b*-poly(lactic-*co*-glycolic acid) copolymers (PLGA–PEG–PLGA) used in this study, PLGA-PEG-PLGA (Mw ∼1,000:1,000:1,000 Da) LA:GA 1:1 (PPP 1000) and PLGA-PEG-PLGA (Mw ∼1,500:1,500:1,500 Da) LA:GA 6:1 (PPP 1500), were purchased form PolySciTech (Akina Inc.). CpG1826 (5′-tccatgacgttcctgacgtt-3′) was purchased from Integrated DNA Technologies, and CpG1826 with 5′ benzaldehyde modification was purchased from TriLink Biotechnologies. Both CpG1826 (CpG) with and without 5′ benzaldehyde modification included nuclease-resisting phosphorothioate (PS) backbone. All other buffers, chemicals, and materials were purchased from Fisher Scientific and used as received unless otherwise mentioned.

### Preparation of PLGA-PEG-PLGA solutions and mixtures

The hydrogels with different ratios of PPP 1000 and PPP 1500 at different concentrations were prepared; PPP 1000 and PPP 1500 were dissolved in PBS, respectively, to obtain a 30% w/v concentration, and stirred at 4 °C for 2 days. The 30% w/v solution of PPP 1000 and PPP 1500 were mixed in different volume-to-volume (v/v) ratios: 100:0, 75:25, 50:50, 25:75 and 0:100. Then the mixtures were further diluted in PBS to achieve concentrations ranging from 5% w/v to 25% w/v.

### Characterization of PLGA-PEG-PLGA hydrogels

#### Rheological characterization of PLGA-PEG-PLGA hydrogels

Rheological properties of the polymer solution mixtures were measured with a rheometer (Discovery HR-3, TA instruments) using a 25 mm parallel plate geometry. A solvent trap was used to minimize water evaporation from the sample. The polymer solution was placed between plates with a gap of 150 µm and was equilibrated to the starting temperature. The storage and loss modulus (G′ and G″) were measured as a function of temperature between 10 and 60 °C with the heating rate of 1 °C/min, which we confirmed was low enough to not impact the extracted results from the rheology experiment. The temperature ramp tests were carried out with a fixed oscillatory frequency of 1 Hz and strain amplitude of 0.1%, which we confirmed was in the linear viscoelasticity region for our hydrogels (Supplementary Figure S3). Four different parameters relevant to our analysis were extracted from the G′ vs. T plot using Trios software (TA Instruments): the peak storage modulus (G′_peak_), the temperature corresponding to G′_peak_ (T_peak_), the onset temperature (T_1_), and the offset temperature (T_2_). T_1_ and T_2_ were calculated by extrapolating tangent lines to G′ at the inflection points on the rising and falling sides of G′_peak_ and measuring the intersection of the tangent with the temperature axis.

#### Transmission electron microscopy (TEM)

The TEM images of copolymer micelles were obtained from a JEM-2100F (JEOL) transmission electron microscope with a Gatan OneView camera (Gatan) at 200 kV. TEM samples of PPP at 25 °C were prepared by placing 10 µL of 1% w/v 25:75 PPP solution on a carbon-coated copper grid and staining with 2% uranyl acetate. TEM samples of PPP at 37 °C were prepared by placing a carbon coated copper grid on top of a 10 µL drop of 1% w/v 25:75 PPP solution incubated at 37 °C, and then staining with 2% uranyl acetate.

#### Dynamic light scattering (DLS)

The hydrodynamic diameter of the PPP micelles was characterized by DLS (Zetasizer Nano ZS, Malvern). A PPP solution of 0.1% w/v in PBS was incubated at 5, 20, 36, and 50 °C for 5 minutes prior to measurement.

### Expression, purification, and characterization of SpyTag-E2 (“ST-E2”) particles, SpyCatcher-CBU1910 (“SC-CBU”), and CBU1910 (“CBU”)

ST-E2 used in this study was derived from a previously reported mutant D381C (Dalmau et al. 2008; Molino et al. 2013, 2016; Neek et al. 2018). The plasmid encoding ST-E2 was constructed via PCR, and ST-E2 was expressed and purified as previously described (Li et al. 2022; Ramirez et al. 2023). In brief, *E. coli* BL21(DE3) containing ST-E2 in a pET11a plasmid was cultured in LB media containing 100 μg/mL of ampicillin at 37 °C. When OD_600_ reached 0.7–0.9, 1 mM IPTG was introduced, and the culture was further incubated for 3 hours. Cells were pelleted and frozen at −80 °C. Cells were lysed using French Press (Thermo Fisher Scientific) in a Tris-based lysing buffer containing 1 mM PMSF. The supernatant of the lysates was heat-shocked at 70 °C and centrifuged again to remove aggregates. The supernatant was purified using a HiPrep Q Sepharose anion exchange column (GE Healthcare) and Superose 6 size exclusion column (GE Healthcare). Lipopolysaccharide (LPS) was removed following a previously established method (Molino et al. 2013). In brief, Triton X114 (Sigma) was added into the protein solution at 1% v/v. The mixture was incubated at 4 °C, mixed using a vortex, heated to 37 °C, and centrifuged at 18,000 × *g* at 37 °C for 1 minute. The supernatant was then carefully collected. This process was repeated 8 times. The final LPS level of the protein (<0.1 EU/µg) was examined with LAL test (ToxinSensor gel clot assay, Genscript). The purified ST-E2 was characterized by DLS (Zetasizer Nano ZS, Malvern), SDS-PAGE, and bicinchoninic acid assay (BCA) as previously described (Molino et al. 2013).

The plasmid encoding SC-CBU was constructed via PCR (“SC” is SpyCatcher and “CBU” is the CBU1910 protein antigen), and the corresponding SC-CBU protein was expressed and purified as previously described (Ramirez et al. 2023). In brief, the *E. coli* BL21(DE3) containing the pET11a-SC-CBU1910 plasmid was cultured in LB media and induced with 1 mM IPTG for 3 hours at 37 °C. The cells were pelleted and frozen at −80 °C. Cells were then lysed by French Press and the supernatant was further purified with HisPur Ni-NTA resin (Thermo Fisher Scientific) following the manufacturer’s protocol. In short, the cell lysates were mixed with equal volume of equilibration buffer and then mixed with the HisPur Ni-NTA resin. Then, the mixture was incubated at 4 °C for 1 hour. The resin was packed and washed with a wash buffer and then eluted with an elution buffer containing 75, 150, and 250 mM of imidazole, respectively. Imidazole was removed by dialysis using a 6–8 kDa MWCO dialysis tubing in PBS. The LPS in the purified SC-CBU was removed following the same procedure as LPS removal of ST-E2. The purified protein was characterized by SDS-PAGE and BCA for purity and concentration. Limulus Amebocyte Lysate (LAL) test was used to measure the endotoxin level (<0.1 EU/µg).

CBU1910 used for vaccination and IgG detection by ELISA was obtained from Genscript (Piscataway, NJ); CBU1910 was expressed in *E. coli* BL21 cells and purified by column chromatography, and endotoxin was removed. The purified CBU1910 was evaluated by SDS-PAGE and BCA assay. Endotoxin concentration (<0.1 EU/µg) was measured using a LAL assay (MPL Laboratories, Sparta, NJ).

### CpG and SC-CBU conjugation onto ST-E2 and nanoparticle (NP) characterization

CpG with a 5′-benzaldehyde modification (aldehyde-CpG) was used for conjugation. The conjugation was performed as previously reported (Molino et al. 2013). In short, the cysteines in the internal cavity of E2 were reduced with TCEP. Then, a 10-fold molar excess of *N*-(β-maleimidopropionic acid) hydrazide (BMPH) linker (to E2 monomer) was added to the mixture and incubated at room temperature for 2 hours, and any excess linker was removed using Zeba desalting columns (ThermoFisher, 40 KDa MWCO). A two-fold molar excess of aldehyde-CpG was added to the ST-E2, incubated at room temperature overnight, followed by passing through a Zeba desalting column to remove unconjugated CpG. Conjugation efficiencies and ratios were quantified by the band intensities on SDS-PAGE gel.

SC-CBU and ST-E2 were supplemented with 0.085% (w/v) Sarkosyl (SLS) to prevent precipitation/aggregation (Ramirez et al. 2023), and the two reactants were mixed with a 0.5:1 (SC-CBU:ST-E2 monomer) molar ratio and incubated at room temperature for 20 hours. SDS-PAGE, BCA, DLS, and TEM were used to characterize the molecular weights, conjugation efficiencies and ratios, concentrations, size, monodispersity, and integrity of the protein NP. TEM images were obtained from a JEM-2100F (JEOL) instrument with a Gatan OneView camera (Gatan).

### *In vitro* release test and protein integrity after release from hydrogel

#### E2 released from PPP

Fluorescently-labeled E2 used for both *in vitro* (E2-AF647) and *in vivo* (E2-AF750) studies were conjugated using the same method. Briefly, E2 was reduced with TCEP for 30 minutes prior to conjugation at room temperature. Maleimide-Alexa Fluor (AF647 or AF750) was dissolved in DMSO and added to the E2 solution, with a 10-molar excess of dye to the E2 monomers. The reaction was conducted at room temperature in the dark for 2 hours with gentle agitation. Excess dye was removed with Zeba Spin Desalting Columns.

To characterize the release rate of E2 NP from PPP gel, E2 fluorescently-labeled with Alexa Fluor 647 (E2-AF647) were loaded into 25:75 (PPP1000:PPP1500) PPP solution to obtain a final E2 concentration of 0.33 mg/mL and PPP concentration of 20% w/v. A mixture of 150 μL was added to cluster tubes, and the tubes were incubated at 37 °C for 30 minutes to allow gel formation. PBS containing NaN_3_ (0.02%, w/v) (300 μL), pre-warmed at 37 °C, was added into the tubes gently as a release media. The tubes were sealed and incubated in a shaking incubator at 37 °C and 50 rpm. On days 1, 4, 7, 14, 21, 28, and 35, 150 μL of release media was withdrawn (for characterization) and replaced with the same volume of pre-warmed fresh media. The amount of E2 released from the gel was characterized by measuring the fluorescence intensity of the release media and calculated with a fluorescence standard curve for E2-AF647 solutions.

#### Protein integrity after hydrogel release

Ovalbumin (OVA) was used as the model protein to characterize the integrity of the released protein from PPP gel; this was because the sensitivity of the circular dichroism measurement required a higher protein concentration which could not be obtained by the E2 NP scaffold without precipitation. OVA was dissolved with PBS and loaded into a PPP solution to a final concentration of 10 mg/mL OVA in 20% w/v PPP solution. The mixture was incubated at 37 °C for 10 minutes, and 300 μL of 37 °C pre-warmed sterile PBS was added into the tubes gently as release media. The tubes were sealed and incubated in a shaking incubator at 37 °C and 50 rpm. On day 4, the release media were collected from the respective samples and centrifuged at 15,000 × *g* for 5 minutes. The primary structure of OVA was characterized by SDS-PAGE and the secondary structure was characterized by circular dichroism (Chirascan V100 spectropolarimeter, Applied Photophysics).

### Mice

All animal studies were carried out under protocols approved by the Institutional Animal Care and Use Committee (IACUC) at the University of California, Irvine (UCI). The laboratory animal resources at UCI are internationally accredited by the Association for Assessment and Accreditation of Laboratory Animal Care (AAALAC #000238). For *in vivo* tests comparing NP release after hydrogel encapsulation vs. bolus injection, four 4–5 week old female SKH1-Elite mice were used. SKH1 mice (Charles River) were selected for this part of the study because they are hairless and albino, which allows for better imaging. Immunization studies were performed in 4–6 week old female C57BL/6 mice (Charles River), with a total of 35 mice (*n* = 5 per experimental group). All animals were purchased from Charles River, and all animals weighed between ∼16–19 g at the initiation of the study.

### *In vivo* release test

To quantify the persistence of NPs at the injection site over time, E2 fluorescently labeled with Alexa Fluor 750 (E2-AF750) was loaded into a 25:75 PPP solution to obtain a final E2 concentration of 0.1 mg/mL and PPP concentration as 20% w/v. This PPP-based mixture (100 μL) was injected to the lower back of SKH1 mice subcutaneously. As a bolus control, an equivalent amount of E2-AF750 was mixed with PBS and injected in the same manner as the PPP group. The mice were anesthetized with isoflurane during imaging and the fluorescence was tracked up to 8 weeks using the *In vivo* Imaging System (IVIS Lumina II, Xenogen) with an excitation laser of 745 nm and emission filter of 810–875 nm. The radiant efficiency of the injection sites with a constant regions of interest (ROI) in the *in vivo* images was quantified using Living Image software (Revvity). The radiant efficiency of the ROI from mice injected with PBS was used as background signal for the respective time point and were subtracted from all the groups. The radiant efficiency of the ROI from the images captured for each mouse right after injection was used as 0% of release to normalize the data from the same mouse, respectively. IVIS imaging used two individual mice and observed tight consistency of the results.

### *In vivo* immunizations

C57BL/6 mice were immunized in the lower back via subcutaneous (s.c.) route on day 0. Groups included PPP (negative control), CBU, CBU with PPP, CBU-CpG-E2, CBU-CpG-E2 with PPP, CBU + CpG with PPP, and CBU-E2 with PPP (Figures 6(A) and 7(A)). Each administration dose with CBU antigen contained 3 μg equivalent amount of CBU per dose in each group with antigen. Each dose in groups CBU-CpG-E2, CBU-CpG-E2 with PPP, and CBU + CpG with PPP contains 0.6 μg of CpG. The vaccine components were dispersed in 20% w/v PPP solution, and the administered volume per dose in the groups with PPP hydrogel was 150 μL. Each dose in the bolus groups (CBU or CBU-CpG-E2 with no PPP hydrogel) was 50 μL and was dissolved in PBS. All the vaccinations were administered via syringes with 28-gauge needles. Blood sera were collected from the submandibular (facial) vein throughout this study.

### ELISA

96-well plates (Thermo Fisher) were coated with 100 μL per well of CBU at 2 μg/mL in 1× TBS (20× from Thermo Fisher, diluted with water before use) at 4 °C for overnight. Plates were washed four times with 300 μL/well 1X TTBS (20× from ChemCruz, diluted with water before use), then blocked with 300 μL/well of 1% casein–TBS (Thermo Fisher) at room temperature for 1 hour. The casein-TBS was removed from the wells, and the plates were air-dried at room temperature. Serum samples were diluted 1:900 with casein–TBS. Diluted serum (100 μL/well) was incubated in the plates for 45 minutes on a shaker, then plates were washed four times with 300 μL/well of 1× TTBS. One of the following secondary antibodies was diluted 1:10,000 and used: Goat anti-mouse IgG Fc-HR, Goat anti-mouse IgG1 Fc-HRP, Goat anti-mouse IgG2c Fc-HRP (Bethyl Laboratories). Diluted secondary antibody (100 μL/well) was incubated in the plate for 45 minutes on a shaker. The plates were washed 4 times with 300 μL/well of 1X TTBS. Plates were developed with 100 μL/well TMB substrate (SureBlue Reserve, KPL, Inc.) for 2 minutes. The reaction was stopped with 100 μL/well 0.18 M H_2_SO_4_. The absorbance at 450 nm for each well was measured with a microplate reader.

### Statistical analyses

Statistical analyses were performed with GraphPad Prism 10. All the hydrogel characterizations were performed minimally in triplicate (*n* ≥ 3), and immunization studies included *n* ≥ 5 individuals per group. One-way or two-way ANOVA followed by Tukey’s multiple comparison test over the experimental groups were used as described in figure captions or unless otherwise specified. *p* < 0.05 was considered statistically significant.

## Results and Discussion

**Figure 1.**
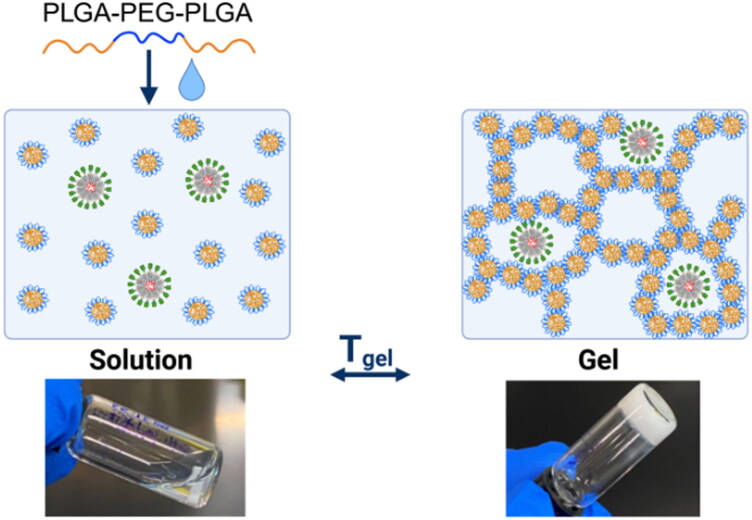
Overview of the injectable nanoparticle-loaded hydrogel vaccine. The nanoparticle-PPP solution is injected at room temperature and forms a sustained-release depot at physiological temperature.

In this study, we examined the effects of a controlled-release strategy on antibody response to a Q fever vaccine. There are two main components of this slow-release vaccine: the E2 protein NP scaffold that co-delivers the *C. burnetii* antigen and a TLR9 agonist (adjuvant), and the hydrogel depot material comprising PLGA-PEG-PLGA (PPP) block copolymers for extended release. The NP is first synthesized and then mixed into the polymer solution at room temperature (Figure 1). This mixture will transition into a hydrogel after injection *in vivo*.

### Synthesis and characterization of nanoparticles (NPs)

**Figure 2.**
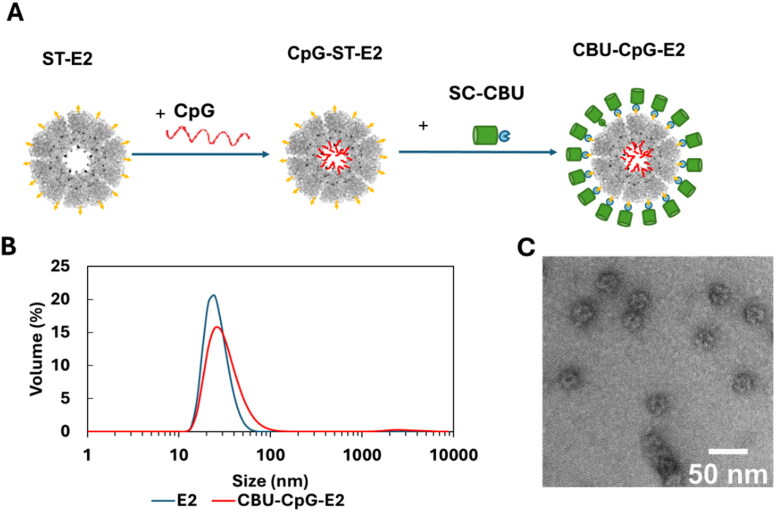
Conjugation of CBU1910 antigen and CpG adjuvant onto E2 nanoparticles. (A) Schematic of vaccine nanoparticle synthesis, with components ST-E2 (SpyTag [ST] in yellow, E2 in grey, and cysteines on E2 in black), adjuvant CpG1826 (red), and SC-CBU (SpyCatcher [SC] in blue and CBU in green). CpG1826 and SC-CBU are conjugated on E2 nanoparticle on the interior and exterior surfaces, respectively, to form CBU-CpG-E2. (B) Representative hydrodynamic diameter of E2 (unconjugated) and CBU-CpG-E2. (C) Representative TEM image of the CBU-CpG-E2 nanoparticle.

We have previously shown that E2 NPs can be used as a platform for the co-delivery of adjuvant and peptide antigen to elicit an antigen-specific anti-tumor response (Molino et al. 2013; 2016; Neek et al. 2018). These studies demonstrated that antigen-bound NPs increased the level of antigen uptake by dendritic cells (DCs), and the simultaneous co-delivery of antigen and adjuvants enhanced DC activation and peptide-specific CD8 T cell responses. As described in our previous study, the E2(D381C) NPs were genetically fused with SpyTag (ST) to enable modular antigen conjugation and were expressed in *E. coli* (Ramirez et al. 2023). The adjuvant, CpG, was conjugated onto the cysteines located at the interior cavity of the SpyTag-fused E2 NPs (ST-E2) (Figure 2(A)). CpG is a TLR9 agonist which can activate DCs and elicit a T helper-1 (Th1) type immune response (Murad and Clay 2009). Our previous study has shown that the E2-conjugated CpG are released under acidic endosomal conditions and activate DCs (Molino et al. 2013).

In this study, *Coxiella* outer membrane protein CBU1910 (CBU), which is also known as Com1, was selected to be used as the antigen because it is an immunodominant antigen that has previously been demonstrated as an effective vaccine target against Q fever (Vigil et al. 2010; Xiong et al. 2012; Gerlach et al. 2017). The protein antigen, CBU, is genetically fused with a SpyCatcher (SC-CBU) and expressed in *E. coli* as described in previous work (Ramirez et al. 2023). SC-CBU was then conjugated with CpG-conjugated ST-E2 (CpG-ST-E2) through the ST/SC reaction, which is advantageous because it presents a uniform antigen orientation, which is important for B cell activation (Ramirez et al. 2023) (Figure 2(A)). The molecular weight and the number of CBU or CpG per NP were confirmed and determined by SDS-PAGE (Supplementary Figure S1) and used to adjust the dosage of the vaccine for immunization. ST-E2 has a molecular weight of 30.2 kDa, and the band at 35 kDa confirms the successful conjugation of CpG on E2. The two bands around 80 kDa shows the synthesis of the final CBU-CpG-E2 NP; the higher band is the monomers of CBU-CpG-E2, and the lower band shows the monomers of CBU-E2 mixed within the CBU-CpG-E2 assembly. Consistent with our previous work, each E2 NP has 24 ± 2 CBU molecules on the exterior surface and 20.5 ± 1.5 CpG1826 on the interior surface (Ramirez et al. 2023). The hydrodynamic diameter and assembly of the final NPs were assessed by DLS and TEM (Figure 2(B, C), respectively). The hydrodynamic diameter of the E2 conjugated with CpG and CBU (CBU-CpG-E2) was 37.1 ± 3.5 nm, which was higher than that of ST-E2 (27.6 ± 0.4 nm); this size increase also supports that the CBU molecules were conjugated onto the E2 NPs (Figure 2(B)). The baseline scaffold, E2 (D381C variant), was used as the control NP for comparison and as the model NP in the subsequent polymer release studies.

### Rheological properties of PLGA-PEG-PLGA mixtures

**Figure 3.**
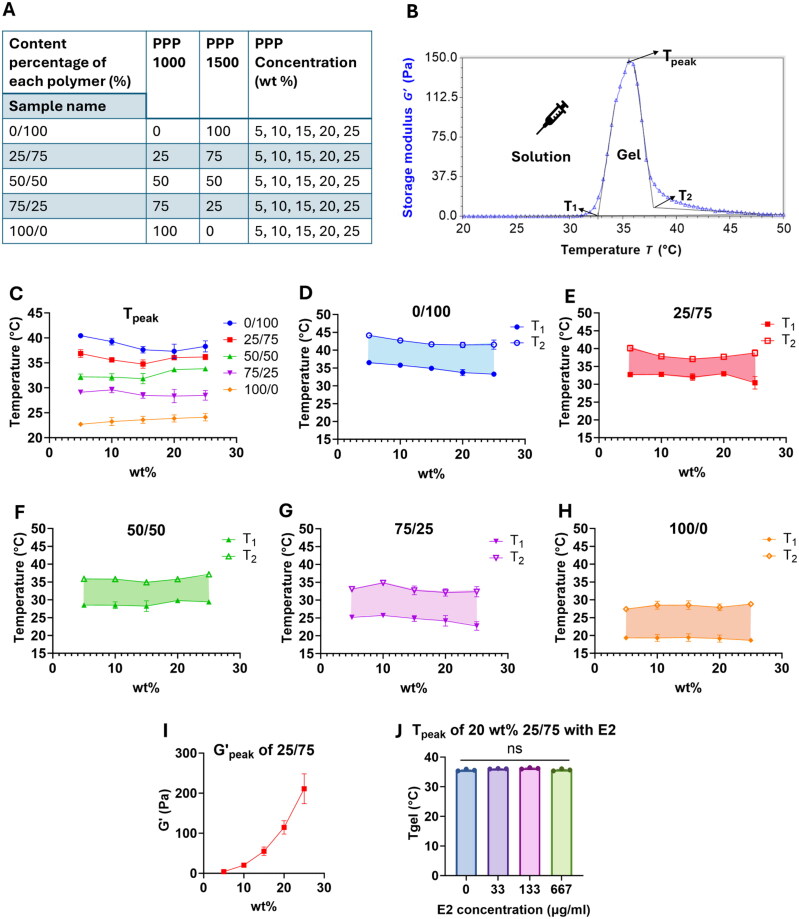
Rheological properties of PPP. (A) PLGA-PEG-PLGA (PPP) mixtures comprise PLGA-PEG-PLGA (Mw 1000:1000:1000 Da) LA:GA 1:1 (PPP 1000) and PLGA-PEG-PLGA (Mw 1500:1500:1500 Da) LA:GA 6:1 (PPP 1500), mixed in different ratios (0/100, 25/75, 50/50, 75/25, 100/0; vol/vol), and at total weight concentrations ranging 5–25 wt%. (B) Representative rheological measurement for PPP 1000/PPP 1500 at a 25/75 ratio, and 20 wt%. shown are the T_1_, T_2_, T_peak_, and the corresponding storage modulus, in which T_1_ and T_2_ are the onset and endset temperatures of storage modulus, T_peak_ is the temperature where the storage modulus G′ is at the maximum. (C) T_peak_ of PPP mixtures at different PPP 1000/PPP 1500 ratios and at different concentrations. (D–H) T1 and T2 of PPP solution mixtures at different PPP 1000/PPP 1500 ratios and at different concentrations. (I) Storage modulus (G′) at T_peak_ of 25/75 PPP mixture at different concentrations. (J) T_peak_ of 20 wt% PPP mixture (PPP 1000/PPP 1500 = 25/75) loaded with model protein E2 at different concentrations. Data points for panels C–J are average ± S.E.M. values of *n* ≥ 3 individual samples.

To be used as an injectable depot for vaccines, the hydrogel material needs to be in a liquid state at room temperature for it to be easily mixed with the protein NP delivery system and injectable with a needle. Subsequently, it needs to acquire viscoelastic properties at the physiological temperature of the subcutaneous region under the dermis in mice, which has been reported to be 35.9–37.5 °C (Vlach et al. 2000). This gel also requires sufficient physical strength (G′ in the range of 10^1^–10^4^ Pa) for the depot to tolerate and resist stress from the movement of the adjacent tissue (Song et al. 2019; Osorno et al. 2020; Yang et al. 2020; He et al. 2021; Wei et al. 2021). Therefore, we fabricated different hydrogel formulations and examined their corresponding rheological characteristics across the relevant conditions (Figure 3).

We examined the characteristics of PPP copolymers with different molecular weights and lactic acid (LA)-to-glycolic acid (GA) ratios for formulating the depot material. Based on the gelation temperatures (Mohammadi et al. 2018; Osorno et al. 2020) and degradation times (Lei et al. 2022) reported in the literature and by commercial vendors, we investigated several PPP copolymers, and polymers PPP 1500 (Mw 1500:1500:1500 Da, LA:GA 6:1) and PPP 1000 (Mw 1000:1000:1000 Da, LA:GA 1:1) were selected for further studies. Based on the dependence of G′ on T from rheological measurements (Figure 3(B)), as the temperature for a solution of PPP 1500 is increased, the solution turns into a hydrogel as characterized by a sharp increase in the mixture’s storage modulus (G′) up to a maximum value (G′_peak._). Further increases in temperature result in a rapid drop in G′ to values comparable to the pre-gelled mixture, which we suggest is due to precipitation of the polymer out of the solution. The values of T_1_, T_2_, T_peak_, and G′_peak_ were extracted from the G′ vs. T plot as described in the “Materials and Methods” section, for all formulations listed in Figure 3(A). The onset temperature (T_1_) for PPP 1500 ranges from 33.3 ± 0.9 °C (for 25 wt%) to 36.5 ± 0.6 °C (for 5 wt%) (Figure 3(D)), the value of T_peak_ ranges from 37.3 ± 2.8 °C (20 wt%) to 40.5 ± 0.5 °C (5 wt%) (Figure 3(C)), while the offset temperature (T_2_) ranges from 41.6 ± 2.6 °C (25 wt%) to 44.1 ± 1.2 °C (5 wt%) (Figure 3(D)). The behavior is similar for the polymer PPP 1000, with T_1_ ranging from 18.6 ± 1.3 °C (for 25 wt%) to 19.3 ± 1.3 °C (for 5 wt%), T_peak_ ranging from 22.7 ± 0.7 °C (5 wt%) to 24.1 ± 1.5 °C (25 wt%), and T_2_ ranging from 28.8 ± 11.0 °C (25 wt%) to 27.4 ± 0.4 °C (5 wt%) (Figure 3(C, H)). These results showed that the individual solutions of PPP 1500 and PPP 1000 were both suboptimal for the conditions needed in our application, as their gelation temperature ranges were at best only marginally within the relevant physiological temperatures.

Previous studies have shown that the gelation temperature of mixtures of two copolymers can be tuned by changing their ratio as well as the overall polymer concentration (Li et al. 2013; Zhang et al. 2015; Sharma et al. 2021; Wei et al. 2021). Thus, to obtain a material with a more optimal gelation temperature range to use as a vaccine depot *in vivo*, we examined PPP mixtures with different ratios of PPP 1000/PPP 1500 and overall polymer concentrations (Figure 3(A)). The T_1_, T_2_, and T_peak_ for these mixtures were measured (Figure 3(B)), and results are presented in Figure 3(D–G). We found that the mixture containing a 25/75 (vol/vol) ratio of 20 wt% solution of PPP 1000/PPP 1500 has a T_peak_ at 36.0 ± 0.9 °C, T_1_ at 32.2 ± 0.5 °C, and T_2_ at 38.2 ± 1.0 °C, temperatures which are better suited to form consistent vaccine depots at the reported subcutaneous temperatures in mice (Vlach et al. 2000). We also observed that the storage modulus (G′) of the gel increases as the concentration of the PPP mixture increases, suggesting that higher concentrations are preferable in terms of physical strength of the hydrogel. However, the final vaccine formulation also included the volume for the antigen-bound NPs and adjuvant in the PPP; with all the requirements and constraints, the final concentration of the PPP we used in this study was 20 wt%, which yielded a G′ of < 0.1 Pa at 25 °C (injectable at room temperature) and 114.7 ± 33.8 Pa at T_peak_ and physiological temperatures. For the remainder of the study, a PPP hydrogel mixture of PPP 1000/PPP 1500 at a 25/75 vol/vol ratio, with a total polymer concentration of 20 wt%, was used.

### Effect of model protein loading on gelation temperature and storage modulus

Vaccine NPs will be embedded within the polymer mixture, so we examined the effects of the protein NPs on the polymer gelation properties to ensure that gel-like properties are maintained, even with the antigen payload. We used the E2 NP scaffold as the model protein, and measured T_peak_ within a protein concentration range which is typically used for a single dose of subunit vaccine antigen conjugated to the NP scaffold (5–100 μg antigen dose in 150 μL injection). PPP was at the ratio and the final weight concentration that was used in further studies (25/75 ratio of PPP 1000/PPP 1500 and 20% PPP, respectively). As shown in Figure 3(J), loading model protein within the concentration range of 0–667 μg/mL does not alter the T_peak_ of the PPP gel. This demonstrates that the PPP gel formulation tested can be used as a gel depot material for a protein-based vaccine of sufficient dosage.

### Sol–gel properties of PLGA-PEG-PLGA (PPP) in aqueous solution

**Figure 4.**
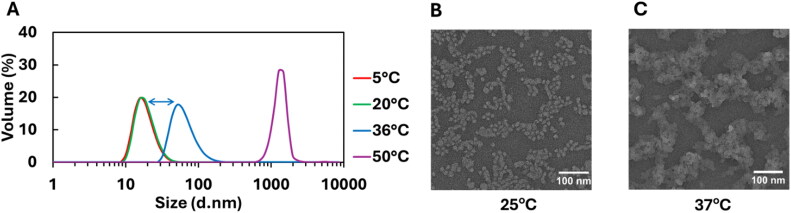
Characterization of PPP micelles and thermal-triggered self-assembly. (A) Hydrodynamic diameters of micelles or assemblies formed in 0.1 wt % PPP solution at 5 °C, 20 °C, 36 °C, and 50 °C, measured with DLS. TEM images of 1 wt % PPP, at (B) 25 °C and (C) 37 °C.

We examined the macromolecular self-assembly of PPP. We studied the formation of micelles and the thermosensitive behavior by characterizing the PPP mixture with DLS and TEM at different temperatures. Figure 4 suggests that the micelles assembled into a gel network when temperature increased from 20 to 37 °C. The hydrodynamic diameters of PPP micelles at 5 °C and 20 °C were measured by DLS to be 19.6 ± 1.6 nm and 19.7 ± 1.4 nm, respectively (Figure 4(A)), and this was supported by TEM images (Figure 4(B)). When the temperature is increased to physiological values (36–37 °C), the micelles appear to form larger clusters, which may suggest the formation of gel networks at higher concentrations, which are consistent with TEM images (Figure 4(B)). At 50 °C, the diameter of the particles becomes much larger, which, in conjunction with the mixture’s near-zero elastic modulus at this temperature (Figure 3(B)), may indicate precipitation of polymers.

Studies of similar PLGA-PEG-PLGA triblock polymers using different methods also showed evidence that the sol-gel transition is due to the assembly of micelles into an interconnected network. Khorshid et al. used small-angle neutron scattering (SANS) to characterize the structural features of a PPP solution and found the diameter of their micelles to be 19.4 nm at 10 °C (Khorshid et al. 2016), which is consistent with our result from DLS. They also demonstrated that the PPP gel network is composed of micelles linked to an irregular transient network (Khorshid et al. 2016). Others have used Monte Carlo simulations to study the assembly of PPP micelles and determined that the gel network forms hydrophobic channels and hydrophilic bridges between micelles (Cui et al. 2018, 2019). The gel structures presented by these previous works are consistent with our TEM images of PPP at 37 °C.

### *In vitro* and *in vivo* release profiles and integrity of protein released from PPP

**Figure 5.**
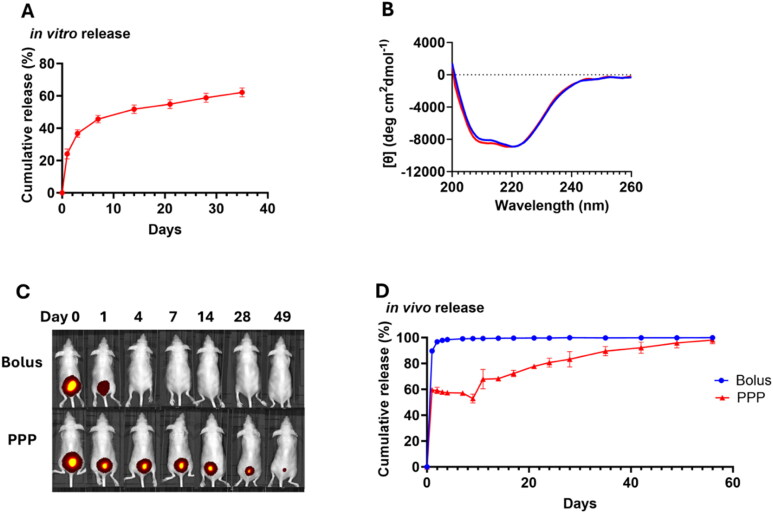
The PPP mixture can serve as a depot material to release protein in a sustained profile. (A) *In vitro* release profile of fluorescently-labeled E2 NP from PPP gel at 37 °C (*n* = 3; average ± SD). (B) Representative circular dichroism (CD) spectra of ovalbumin (OVA) released *in vitro* from PPP gel measured on day 4. (C) Representative images of AF750-E2 NP remaining at the injection site *in vivo*, after injection with formulations that were encapsulated in PPP and as a bolus administration (control). (D) *In vivo* release profile of fluorescently labeled E2 NP, quantified by IVIS. Each data point is shown as average ± SD (*n* = 2).

We examined the release profile of the E2 NP scaffold as a model protein that was encapsulated into the PPP hydrogel. Both *in vitro* (Figure 5(A)) and *in vivo* (Figure 5(C, D)) studies show that this protein experienced a burst release on day 1 and was continuously released from the hydrogel depot in the following 7–8 weeks. We also compared the PPP depot to a conventional bolus administration *in vivo* (Figure 5(C, D)), and found that the bolus injection maintained the protein in the injection site for only 1 day. This showed the potential of the PPP gel for extended release of the protein NP vaccine.

One concern of using a hydrogel depot is the resulting integrity of the released protein vaccine, as antigen protein structure is important for generating a robust humoral immune response (Adams et al. 2015; Chen et al. 2022). To investigate this, we performed an *in vitro* release study using ovalbumin (OVA). We used OVA as the model protein at 10 mg/mL (mixed with PPP in PBS) because OVA can be solubilized and released at a sufficiently high concentration for detection by circular dichroism (CD) spectroscopy, which can evaluate changes in protein secondary structure and folding. As shown in Figures 5(B), OVA released from the PPP hydrogel showed CD spectra with two minima at 208 and 222 nm, which are characteristic of a protein with intact α-helical structure (Greenfield 2006), supporting the integrity of correct protein folding. These spectra are also comparable with those from OVA control solutions (no PPP). SDS-PAGE (Supplementary Figure S2) also shows that the primary structure of the protein did not undergo degradation after encapsulation and release from PPP.

### Hydrogel encapsulation of nanoparticle vaccines enhanced antigen-specific IgG, IgG1, and IgG2c after a single administration

**Figure 6.**
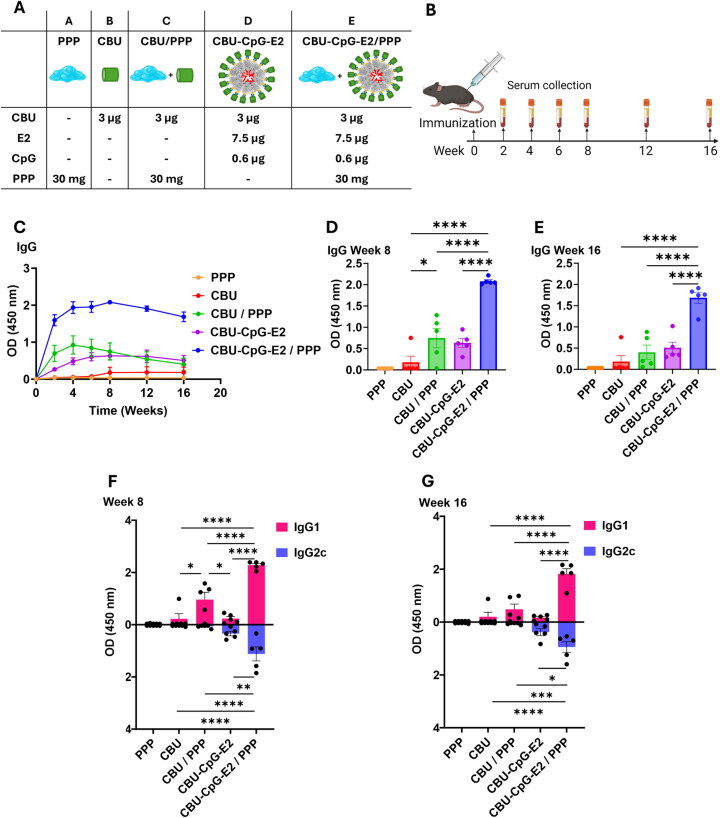
Antibody responses of nanoparticle-loaded hydrogel vaccine. (A) Table of vaccine formulation of each group and their components. (B) Vaccination and serum collection schedule. (C) Total CBU-specific IgG in serum over time (durability). (D, E) Total CBU1910-specific IgG in serum collected at 8 weeks and 16 weeks after vaccination. Each dot represents a biological replicate (*n* = 5 mice). (F, G) CBU-specific IgG1 and IgG2c in serum at week 8 and week 16 after vaccination. Each dot represents a biological replicate (*n* = 5). Data in panels C, D, E, F and G are presented as an average ± SEM of 5 mice per group. Statistical significance was determined by one-way ANOVA followed by a Tukey’s multiple comparisons test. *p < 0.05, **p < 0.01, ***p < 0.001, ****p < 0.0001.

**Figure 7.**
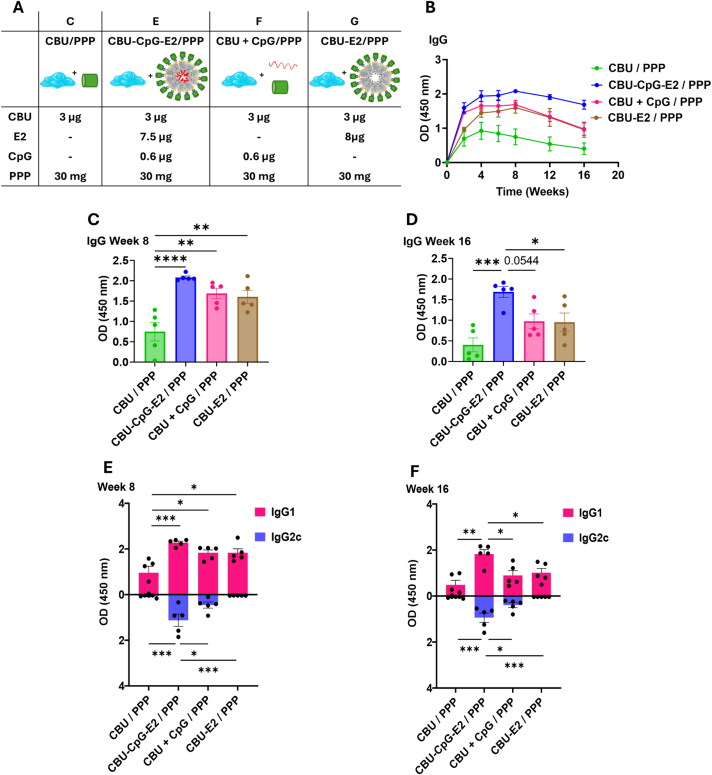
Antibody response of nanoparticle-loaded hydrogel vaccine. (A) Table of vaccine formulation of each group and their components. (B) Total CBU-specific IgG in serum over time (durability). (C, D) Total CBU-specific IgG in serum collected at 8 weeks and 16 weeks after vaccination. Each dot represents a biological replicate, *n* = 5. (E, F) CBU-specific IgG1 and IgG2c in serum at week 8 and week 16 after vaccination. Each dot represents a biological replicate, *n* = 5. Data in panels B, C, D, E, and F are presented as the average ± SEM of 5 mice per group. Statistical significance was determined by one-way ANOVA followed by a Tukey’s multiple comparisons test. *p < 0.05, **p < 0.01, ***p < 0.001, ****p < 0.0001.

We evaluated the humoral immune response of the NP vaccines encapsulated into the PPP hydrogel. Mice were immunized with a single dose of CBU antigen in different formulations as listed in Figures 6(A) and 7(A), according to the immunization schedule presented in Figure 6(B). Overall, as shown in Figure 6(C), the NP-loaded hydrogel vaccine formulation (Group E: CBU-CpG-E2/PPP) elicited the highest IgG at all time points compared with the free antigen (Group B: CBU), antigen-loaded hydrogel (Group C: CBU/PPP), and NP vaccine alone (Group D: CBU-CpG-E2) over all time points. The vaccines formulated with the NPs (Group D: CBU-CpG-E2, Group E: CBU-CpG-E2/PPP) showed higher average total IgG levels than the respective groups containing the free antigen (Group B: CBU and Group C: CBU/PPP), regardless of whether they were encapsulated in PPP gel or not encapsulated. We note that no adverse events or morbidity was associated with immunizations (Supplementary Figure S4).

One of the hypothesized benefits of using a hydrogel to extend the antigen exposure time is a more durable immune response relative to bolus administration (i.e. no hydrogel). Our data shows evidence to support this; at both week 8 and week 16 (Figure 6(D, E)), which represent middle- and long-term antibody responses, respectively, the CBU-CpG-E2/PPP group demonstrates a significantly higher IgG signal than the bolus group (Group D: CBU-CpG-E2). For the antigen groups not conjugated to E2 or CpG, the IgG response was also significantly higher for the CBU antigen embedded in polymer (Group C: CBU/PPP) than for the free CBU group (Group B: CBU) at the early and middle time points (Figure 6(C, D)); however, IgG levels decayed quickly and by week 16, the advantage of using hydrogel depot for this comparison group (CBU antigen without NP) was no longer significant (Figure 6E). These results suggest that the dual aspects of co-delivery of antigen and adjuvant via the NP scaffold, together with the extended release by the hydrogel, are both necessary for eliciting a durable immune response.

IgG2c and IgG1 were quantified as proxy indicators of Th1 and Th2 responses, respectively (Figure 6(F, G)). The CBU/PPP group had a significantly higher IgG1 signal than the free CBU group on week 8, whereas the IgG2c was not higher, suggesting that without the presence of the Th1-skewed adjuvant (e.g. CpG), the hydrogel is Th2-biased. Interestingly, when the complete NP formulation (CBU-CpG-E2) was delivered using the PPP hydrogel, not only did the IgG1 response increase but the IgG2c response also increased in both middle- and long-term timepoints. This result suggests that PPP can enhance the amount of IgG2 that already exists due to the vaccine but does not generate this response on its own. Furthermore, co-delivery of CpG and antigen on a NP scaffold may be necessary for the hydrogel sustained-release vaccine to obtain a more balanced level of Th1 vs. Th2 response.

CD4 (Th1) and CD8 T cells play a central role in host immunity against *C. burnetii* through IFNγ and perforin killing of invaded macrophages, respectively, particularly against the more lethal chronic form. However, antibodies also contribute to protective immunity (although to a lesser extent). Antibodies may play a role in opsonization and neutralization of extracellular bacteria during the early stages of infection but require help from T cells to clear the infection. Therefore, an effective vaccine strategy should elicit both antibody (Th2) and T (Th1) cell responses.

### Sustained co-delivery of antigen and adjuvant elicited high antibody levels over time

The CBU+CpG/PPP group was used to determine the effects of the adjuvant, and the CBU-E2/PPP group was used to evaluate the effects of conjugating the antigen on a NP scaffold in the same experiment (Figure 7(A)). The same immunization dose and serum collection schedule was administered for these two groups, and the IgG on week 8 and week 16 were compared with the CBU/PPP and CBU-CpG-E2/PPP groups. On week 8, the IgG level of the immunization groups CBU-CpG-E2/PPP, CBU+CpG/PPP, and CBU-E2/PPP were all higher than the CBU/PPP group, which suggests that both the presence of CpG and conjugating the antigen on the NP helps to elicit a higher IgG in the early stage after vaccination (Figure 7(C)). However, at week 16, the IgG levels of the CBU+CpG/PPP and CBU-E2/PPP groups decreased more than the CBU-CpG-E2/PPP group, with the latter showing a significantly higher IgG than the CBU-E2/PPP group and a 1.7-fold higher amount of IgG relative to the CBU+CpG/PPP group (*p* = 0.054) (Figure 7(D)).

These data support that the co-delivery of antigen and adjuvant on the NP scaffold in a extended period may be key to eliciting a durable antibody response. We speculate that there are several characteristics of the CBU-CpG-E2 NP vaccine that could contribute to higher immune responses compared to free CBU antigen: (1) the size of E2 NPs conjugated with antigen and adjuvant falls within the optimal size range (20–50 nm) for DC uptake and retention in lymph follicles (Nguyen and Tolia 2021), which will increase antigen and adjuvant uptake; (2) the CpG molecules conjugated on E2 NPs can be released at acidic endosomal conditions and activate DCs by binding to the TLR9 located in endosomal compartments (Molino et al. 2013); and (3) the antigen multivalency on the NP vaccine, exposes each APC to a higher number of CBU1910 molecules than for unconjugated antigen formulations.

The Th1/Th2 skewing of immunized mice was evaluated by measuring IgG2c and IgG1, respectively (Grødeland et al. 2015). Introducing free CpG into the PPP gel helped to elicit a greater Th1 response when comparing CBU/PPP with CBU+CpG/PPP (Figure 7(E, F)). However, the NP co-delivery group (CBU-CpG-E2/PPP) elicited the strongest IgG2c response in both middle- and long-term when compared with the CBU+CpG/PPP group (Figure 7(E, F)); this suggests that with sustained delivery, conjugating the antigen and adjuvant onto the NP scaffold helps promote a stronger Th1 response, likely because both antigen and adjuvant are delivered to the DCs at the same time (Molino et al. 2013). The comparison of IgG1 of week 8 and week 16 suggests that the simultaneous co-delivery of antigen and adjuvant helps to obtain a more durable Th2 response and a more balanced Th1 vs. Th2 response.

In adoptive transfer studies of immune serum, IgG has been shown to provide some protection against infection, perhaps during the early stages, but does not fully control bacterial dissemination and growth (Zhang et al. 2007). Therefore, antibody responses, primarily IgG1, are considered contributory but dispensable for protection against Q fever, and may not be a sole correlate to vaccine efficacy against *C. burnetii*. T cells, however, play a more pivotal role in protection. Both CD4 and CD8 T cells appear to play a role in immunity to *C. burnetii* as immunodeficient mice lacking T cells reconstituted with either CD4 or CD8 T cells are able to control infection (Read et al. 2010). In summary, an effective vaccine for *C. burnetii* will likely need to elicit both humoral immunity to enhance vaccine efficacy by neutralizing bacteria prior to infection of host cells and strong cell-mediated memory from both CD4 and CD8 T cells to control intracellular replication and bacterial clearance.

## Conclusions

We demonstrated the advantages of a modular NP-loaded hydrogel vaccine platform for sustained co-delivery of antigen and adjuvants. The single-dose Q fever vaccine that we developed using this platform showed a stronger and more durable humoral immune response than the soluble bolus NP vaccine or free antigen and adjuvant-loaded hydrogel. The incorporation of the NP into the hydrogel also elicited a more balanced IgG1/IgG2c compared with the vaccine without the co-delivery of antigen and adjuvants. Both factors, the sustained delivery kinetics of the PPP material and the co-delivery of antigen and adjuvants on a NP platform, contributed to the improved humoral immune response. This modular platform is flexible and allows variations in the antigen, adjuvants, and release time (Zentner et al. 2001; Yu et al. 2012; Dutta et al. 2020), which broadens its potential use. Furthermore, the ability to extend antigen release over a longer period of time could enable conventional prime-boost vaccination to be alternatively administered as single-dose vaccines, which would be advantageous for increasing patient compliance and global accessibility while decreasing vaccination costs.

Because of its versatility, this platform can be easily used with other immunodominant proteins of *C. burnetii*, such as heat shock protein GroEL, the outer membrane chaperone OmpH, and the surface protein YbgF (Stellfeld et al. 2020), or a combination of multiple antigens. A study has shown that multivalent vaccines, which contains multiple protein antigens, are more immunogenic than monovalent vaccines (Jan et al. 2023). In addition, this nanoparticle/hydrogel vaccine platform could also be applied to other infectious diseases, such as influenza, HIV, and SARS-Cov-2.

## Supplementary Material

Supplemental Material

## Data Availability

The data supporting this work are accessible upon reasonable request from the corresponding author.
