# Supplementary material for: Development of a single-dose Q fever vaccine with an injectable nanoparticle-loaded hydrogel: effect of sustained co-delivery of antigen and adjuvant
Source: Drug Deliv. 2025 May 2;32(1):2476144. doi: 10.1080/10717544.2025.2476144 (PMC12051587; doi:10.1080/10717544.2025.2476144)
Supplement: Supplemental Material [file IDRD_A_2476144_SM6630.pdf]

SUPPORTING INFORMATION

Development of a single-dose Q fever vaccine with an injectable nanoparticle-loaded hydrogel: effect of sustained co-delivery of antigen and adjuvant

Lu Wang[1], Aaron Ramirez[1], Jiin Felgner[2], Enya Li[1], Jenny E. Hernandez-Davies[2], Anthony E. Gregory[2], Philip L. Felgner[2,5], Ali Mohraz[1], D. Huw Davies[2,5], Szu-Wen Wang[1,3,4,5,*]

[1]Department of Chemical and Biomolecular Engineering

[2]Vaccine Research and Development Center, Department of Physiology and Biophysics

[3]Department of Biomedical Engineering

[4]Chao Family Comprehensive Cancer Center

[5]Institute for Immunology

University of California, Irvine, CA 92697, USA

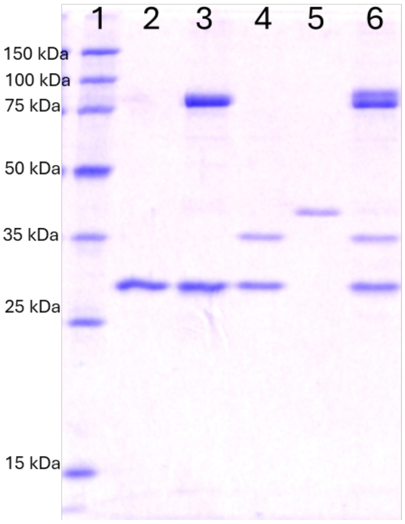

Lane	Sample
1	Ladder
2	ST-E2
3	CBU1910-E2
4	ST-E2-CpG
5	SC-CBU1910
6	CBU1910-E2-CpG

Figure S1. SDS-PAGE of CBU1910-E2-CpG and the conjugation intermediates. Theoretical molecular weight (Mw) of each band in lanes 2-6 and the molecule they represent are listed below. Lane2: ST-E2, (theoretical Mw: 30.2 kDa); lane3: (from top to bottom) CBU1910-E2 monomers (theoretical Mw: ~71 kDa) and ST-E2 monomers on CBU1910-E2 nanoparticles; lane 4: (from top to bottom) ST-E2-CpG (theoretical Mw: ~36.9 kDa) monomers and ST-E2 monomers on ST-E2-CpG nanoparticles; lane 5: (from top to bottom) SC-CBU1910 (theoretical Mw: ~40.8 kDa);lane 6: (from top to bottom) CpG-E2-CBU1910 monomers (theoretical Mw: ~77.6 kDa), CBU1910-E2 monomers, ST-E2-CpG monomers, and ST-E2 monomers on CBU1910-E2-CpG nanoparticles.

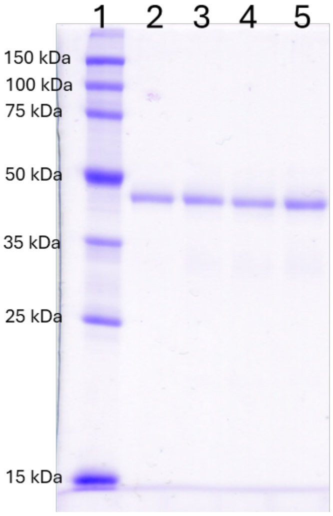

Lane	Sample
1	Ladder
2	Sample #1
3	Sample #2
4	Sample #3
5	OVA control

Figure S2. SDS-PAGE of samples after *in vitro* release of OVA from PPP hydrogel. Samples were collected on day 4 (lanes 2-4) for n=3. OVA solution in PBS was incubated at 37°C for the same duration as the release test samples (control, lane 5).

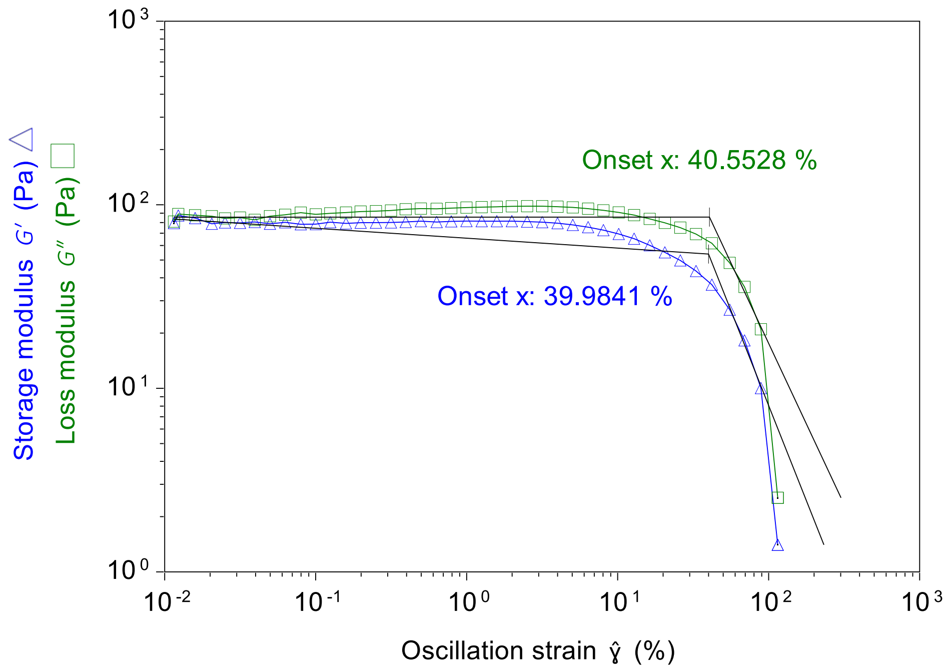

Figure S3. Amplitude sweep for PPP1000/PPP1500 25/75 (v/v), 20 wt% sample. Measurement was performed at 37°C, 1 Hz frequency, G' and G'' were measured as a function of oscillation strain in the range of 0.01% to 100%.

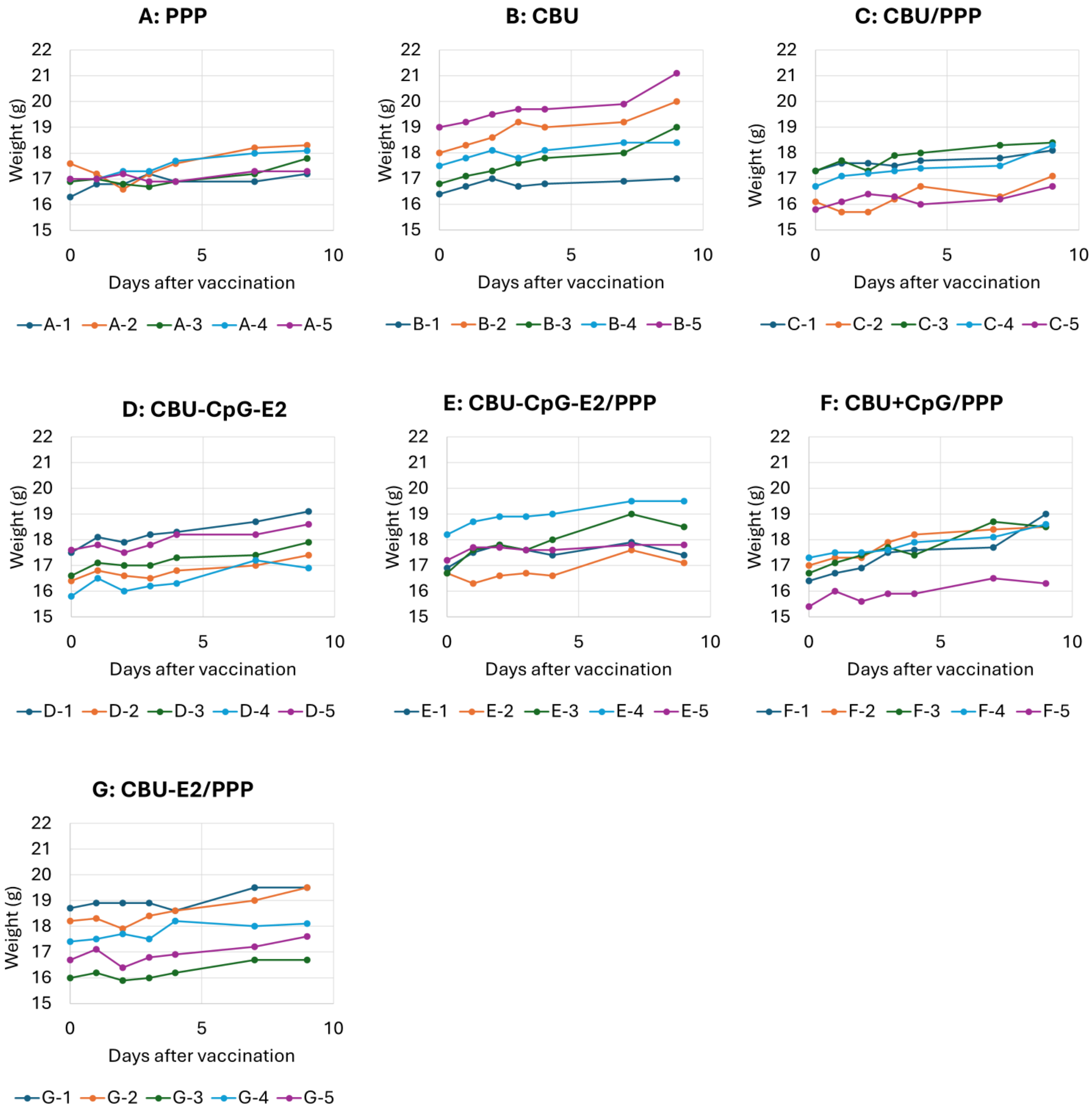

Figure S4. Weight of mice in each group of the study, measured on the day of vaccination and day 1, 2, 3, 4, 7, and 9 after vaccination.
